# School Children’s Physical Activity and Preferred Activities during Outdoor Recess in Estonia: Using Accelerometers, Recess Observation, and Schoolyard Mapping

**DOI:** 10.3390/children10040702

**Published:** 2023-04-10

**Authors:** Getter Marie Lemberg, Eva-Maria Riso, Ingunn Fjørtoft, Lise Kjønniksen, Merike Kull, Evelin Mäestu

**Affiliations:** 1 Institute of Sport Sciences and Physiotherapy, Faculty of Medicine, University of Tartu, 51008 Tartu, Estonia; 2 Department of Sports, Physical Education and Outdoor Studies, Faculty of Humanities, Sports and Educational Science, University of South-Eastern Norway, 3679 Notodden, Norway

**Keywords:** schoolyard, outdoor recess, physical activity, school children, affordances, accelerometer, MVPA

## Abstract

Studies about recess have found that children have higher physical activity (PA) during outdoor recess compared to indoor recess, and well-constructed schoolyards play an important role in stimulating PA in children. This study aimed at investigating the affordances of schoolyards and outdoor recess PA in two urban and two rural primary schools in Estonia. Schoolyards were described with the geographical mapping method, children’s activities during outdoor recess were registered by using observations, and PA levels were measured with accelerometers. Students from grades two to six (8–13-year-olds) were included in the study. All observed schoolyards had different spaces including various ball game areas, climbing facilities, and slacklines. The natural environment dominated in the rural schools, and artificial surfaces dominated in the urban schools. Boys in the study tended to enjoy more sport-related activities, whereas girls preferred more social and less active activities. Students participating in outdoor recess spent about twice as much time (20.4%) on moderate-to-vigorous PA (MVPA) compared to indoor recess (9.5%), although boys were more active than girls (22.9% vs. 17.3%). All schoolyards afforded more MVPA during outdoor recess compared to indoor recess, whereas schoolyards with more space per child and natural environment elements generated more varied PA and higher MVPA. These findings confirm the importance of schoolyard design and quality for the variety and intensity of students’ PA during outdoor recess.

## 1. Introduction

Physical activity (PA) should be part of every child’s everyday life. According to the World Health Organization’s (WHO) recommendations, every child and adolescent (5–17 years old) should be moderately to vigorously active for at least 60 min per day [1]. Regular engagement in PA affects a child’s development [2], can prevent multiple health issues, including obesity and cardiorespiratory diseases [1,3], has a positive effect on academic skills, for example, attention and memory [4,5], and can improve adolescents’ mental health [6].

Studies have found that Estonian children have insufficient PA levels, where approximately 60% of children and adolescents do not meet the global PA recommendations [7]. Furthermore, 80% of children and youth are in front of screens for more than two hours a day during their leisure time [8]. The level of PA decreases and sedentary time increases with age, although boys tend to be more active than girls [9]. Excessive sedentary time increases the risk of chronic diseases regardless of overall PA levels [10].

As children spend most of their waking hours at school, it is important to provide health-enhancing levels of PA opportunities during the school day [11,12,13]. Studies have shown that schools, and especially recess time, have the potential to increase children’s everyday moderate-to-vigorous PA (MVPA) [14,15,16]. Studies that have compared PA levels during indoor recess and outdoor recess have found that children have higher PA levels during outdoor recess [17,18]. In addition, challenging, varied, and exciting schoolyards make children engage more in PA during outdoor recess [15,19]. Holmes [20] and Powell et al. [21] have found that boys and girls have different preferences about what the schoolyard should look like, but most children enjoy natural spaces [22,23,24,25], markings on the ground [26,27], and different types of playgrounds [28]. A recent study in Norway found that both the natural forest and the multivariate constructed schoolyard support the accumulation of MVPA in children [29]. Participating in outdoor recess also has a positive effect on children’s attention, school fatigue, relationships between children and between students and teachers [30], social well-being [31], and academic skills [32,33].

The systematic review by Bikomeye et al. [34] found that including more greenery and natural environment elements in the schoolyard can help to reduce PA equity gaps by providing more play opportunities for females, who tend to engage in less PA compared to their male counterparts, who enjoy competitive play on hardscapes. Raney et al. [24] found that girls spent significantly more time in MVPA during recess after the schoolyard greening process. Therefore, schools need to have diverse schoolyards that offer PA opportunities for every student. Multiple countries around Estonia, like Latvia, Lithuania, Finland, and Norway, have several national policy guidelines regarding schoolyard size, space, and equipment within the schoolyard; however, none of these regulations exist in Estonia [35]. An example of a common schoolyard in Estonia includes mainly flat landscape, some sports fields (basketball, soccer, or track and field), some natural grass and vegetation, and some asphalt as well [35]. Schoolyards also need to be safe for students to use, for example, there should be enough lighting for darker periods, and teachers or supervisors need to oversee the students especially during recess for students from lower school levels.

The Schools in Motion program in Estonia has successfully expanded from 10 pilot schools in 2016 to 184 schools in 2022, providing an opportunity for children to engage in PA and reduce sedentary time during academic lessons and recesses by renewing the physical education curriculum and encouraging changes to the indoor and outdoor environment [36]. One opportunity to be more active during recess is to go outside and use outdoor facilities, which raised the question of outdoor infrastructure and affordances. In co-operation with architects, supportive materials were developed [37]. Implementing outdoor recess in a daily curriculum is a great challenge for schools, and thus, the change takes time.

In Estonian schools, the tradition of outdoor recess is fairly new, and it is not very common to be outdoors during recess, especially in autumn and winter, as the weather conditions are cool and wet for most of the academic year and require appropriate clothing. Most schools allow their students to go outside during recess; however, only some schools have included longer outdoor recess in their daily curriculum. The Schools in Motion program supports outdoor recess as part of every school day. The main goal is for children’s opportunity to go outside during the school day and schoolyards that afford activities to all children with different skills, interests, ages, and genders to be part of the national strategy [7].

Gibson’s theory of affordances describes how the physical environment provides context for human behaviour [38]. “Affordances” are relational characteristics of the physical environment in context with an individual, constituting an action-related context between the person and the environment [39]. The physical environment can encourage various PA behaviour depending on the affordances of the specific environment. Affordances of an environment can be potential and/or actualized [38]. Potential affordances indicate all possibilities that an environment offers, e.g., open space can afford running and jumping, and a tree affords climbing. Actualized affordances refer to the context between the physical environment and the child: it is a child’s response to a certain environment and is visualized through specific types of actions, depending on the child`s perceptions and use of the different affordances.

Some schools in Estonia are moving towards including an outdoor recess in their daily curriculum. Often schools face the question of the quality and affordances of the schoolyard, what facilities and equipment the schoolyard may provide, and what the students would like to do in their schoolyard. This study focuses on four selected schoolyards and their affordances for physical activity during recess.

The main aim of this study was to explore and describe the selected schoolyards and their affordances for PA, and to measure PA during outdoor recess among primary school students in Estonia. The following research questions were examined:

1. What areas, landscapes, and facilities characterize the selected schoolyards, and what are potential affordances in the observed schoolyards?
2. What areas and facilities of the schoolyard do the children use most often (actualized affordances) in outdoor recess?
3. How physically active are the children during outdoor recess in the schoolyards?

## 2. Methods

### 2.1. Participants and Collection of Data

The study included four primary schools in Estonia, and all schools were part of the Schools in Motion program. Two schools were in a more urban setting (Schools A and B) whereas two schools had a more rural setting (Schools C and D). Urban schools were located in cities and rural schools were located in smaller areas with a population of less than 6000. School B and School D were two of the biggest schools, with a total of 909 and 767 pupils, respectively. School A and School C were smaller schools, with a total of 216 and 327 pupils, respectively. Based on set criteria—schools had to have at least 20 min of outdoor recess in their daily curriculum, students had to actively participate in outdoor recess, and schools had to be from different regions in Estonia and have different types of schoolyards—selected schools from various regions in Estonia were invited to participate in the study.

Data collection was performed in autumn 2021 and spring 2022. All students from school level I (grades one to three; 7–10-years-old) and school level II (grades four to six; 11–13-years-old) from participating schools were invited to participate in the study, except for students from grade one, who were excluded from the study due to their different daily curriculum and their difficulty in completing the questionnaire on their own. Written informed consent was obtained from all participants and their legal guardians. All monitoring, collection of data, and analysis were treated anonymously and in line with ethical guidelines. The study was performed in accordance with the Declaration of Helsinki [40] and was approved by the Medical Ethics Committee of the University of Tartu, Tartu, Estonia, approval no. 330/T-7.

### 2.2. Geographical Mapping of the Schoolyards

Schoolyards were described by using a geographical mapping method, showing the design, characteristics, and facilities of the schoolyards. A registration form was used for mapping the schoolyard environments by identifying the space, facilities, equipment, and landscape characteristics of every schoolyard [35,41]. The Republic of Estonia Land Board database [42] was used as the map source. Orthophoto maps [42] were used to describe and identify the schoolyards and facilities for PA. Mapping results were processed with Adobe Illustrator [43]. The size of the schoolyard area was calculated directly from the orthophoto map. School buildings and parking areas were extracted from the schoolyard area.

### 2.3. Describing Potential Affordances for PA in the Schoolyards

Based on Gibson’s theory of affordances [38], the potential affordances of the four schoolyards were described by using the registration form for field observations [35,41]. The schoolyard affordances registration form focused on areas, equipment, facilities, and landscape design for various activities (e.g., ball games, climbing, running, riding, etc.). Based on these registrations, the schoolyards were assessed as to whether they had facilities, equipment, and nature in the schoolyard that stimulate PA.

### 2.4. Outdoor Recess Observation

Outdoor recesses were observed by applying the SOPLAY system for observing play and leisure in youth [44]; however, as observing the PA levels was not the aim of this study, the SOPLAY observation system was adjusted to just observe the behaviour, activities, and facility usage of the students. Hence, during the observation period, all students in the schoolyard were included in the outdoor recess observation, not just the ones who wore the accelerometers. Researchers observed the recess at each school on two consecutive days during the same outdoor recess. The first observation took place on the same day as when students received their accelerometers. After two days of observations, students continued to wear their accelerometers to outdoor recess and filled out an accelerometer diary where they could mark if they participated in the outdoor recess or not. Observations took place in autumn for Schools A and B, and in spring for Schools C and D. School A had two outdoor recesses, one for school level I and the other for school level II, and the recesses lasted 50 min and 30 min, respectively. School B also had two outdoor recesses lasting for 20 min each, but they were not specified for certain grades. Schools C and D had one outdoor recess lasting for 40 min and 30 min, respectively. All students in all four schools had the opportunity to go outside during outdoor recess. Schoolyards were divided into equal-sized areas for researchers to observe. Researchers rotated the areas they observed in each school during the two days of observations. Observers took field notes throughout the outdoor recess and compared their notes at the end of each observation. Later, one of the researchers systematically analysed the field notes to draw conclusions about the facilities and equipment used most in each of the schoolyards. The observation method was piloted at two schoolyards in spring 2021. During the pilot, observed areas were rotated between four researchers, two researchers observed the same area at the same time, and field notes were compared after the recess to assure inter-rater reliability.

### 2.5. Questionnaire

Participants completed a questionnaire including questions about demographics and outdoor recess. The questionnaire was developed and validated by the Schools in Motion program and has been previously used in other studies conducted by them [36]. The demographics section included age, gender, and grade the child was attending. The question about outdoor recess included in the data analysis of this article was “What are three of your favourite activities to do during outdoor recess?” Every student was able to give three different answers to this question and for the results, it was counted as how many times one specific activity was mentioned by students responding to the questionnaire. Responses were grouped based on similar activities and 17 subgroups were formed. For example, different ballgames like basketball, soccer, and table tennis were combined into sport games subgroups. Swings, trampolines, bridge, and tunnels were all grouped as facilities. Climbing on monkey bars, adventure trails, or just climbing was combined into a climbing subgroup. Sitting, playing on the phone, drawing, playing board games, etc., were all grouped as sedentary activities.

### 2.6. Physical Activity

The ActiGraph GT3X accelerometer (ActiGraph LLC, Pensacola, FL, USA) was used to monitor PA and sedentary time during waking hours. Participants were asked to wear the accelerometer on the hip for 7 consecutive days except during water-related sports and activities. A valid recording for PA and sedentary time required at least 4 days (including three schooldays and one weekend day) of at least ten hours of wear time per day. Non-wearing time (at least 20 min of consecutive readings of zero counts and the night-time periods when the unit was removed) was eliminated from the analysis. Data were analysed using ActiLife software version 6.13.4 (ActiGraph LLC, Pensacola, FL, USA). Physical activity intensity zones and sedentary behaviour was calculated based on Evenson et al.’s [45] cut-off points for children. In addition, all children filled out an accelerometer diary where they noted for each measurement day whether they participated in outdoor recess or not. The number of children indicating participation in outdoor or indoor recess was counted for each day and was summed as total participation in outdoor or indoor recess. If the student had not marked participation in outdoor recess or was absent from school, their result was not considered in the analysis. In addition to the accelerometer diary, schools provided their daily curriculum including outdoor recess time. Answers from the accelerometer diary and information from the daily curriculum were connected to accelerometer data to acquire outdoor recess PA data.

### 2.7. Statistical Analysis

Normality of the data was tested using the Shapiro–Wilk test. A Univariate General Linear Model was conducted to examine differences in sedentary behaviour, PA time, and preferred activities by gender, whether one did or did not participate in outdoor recess, and school levels (I and II). All the analyses were adjusted for the length of the recess. SPSS software for Windows (version 28) was used to perform the analyses. Statistical significance was set at *p* < 0.05.

## 3. Results

### 3.1. Schoolyard Mapping and Affordances Registration

The total area of the schoolyards varied between 4581 and 39,905 m^2^, leaving a space of 17–122 m^2^ per child. School C had the largest schoolyard, providing the most space per child. The schoolyards had generally open space and mostly flat landscapes; however, schools A and C had an artificial hill in the schoolyard. All schoolyards had some type of vegetation, but natural grass dominated rural schools, whereas artificial surfaces dominated urban schools. Each observed schoolyard had a slackline, opportunities for ballgames in the schoolyard, and climbing opportunities; however, only urban schools had a full basketball court, and school C was the only schoolyard with the opportunity to play and climb in a natural environment. The four schoolyards had many similarities and differences described in more detail in Table 1 and in the text below.

**Figure 1:**
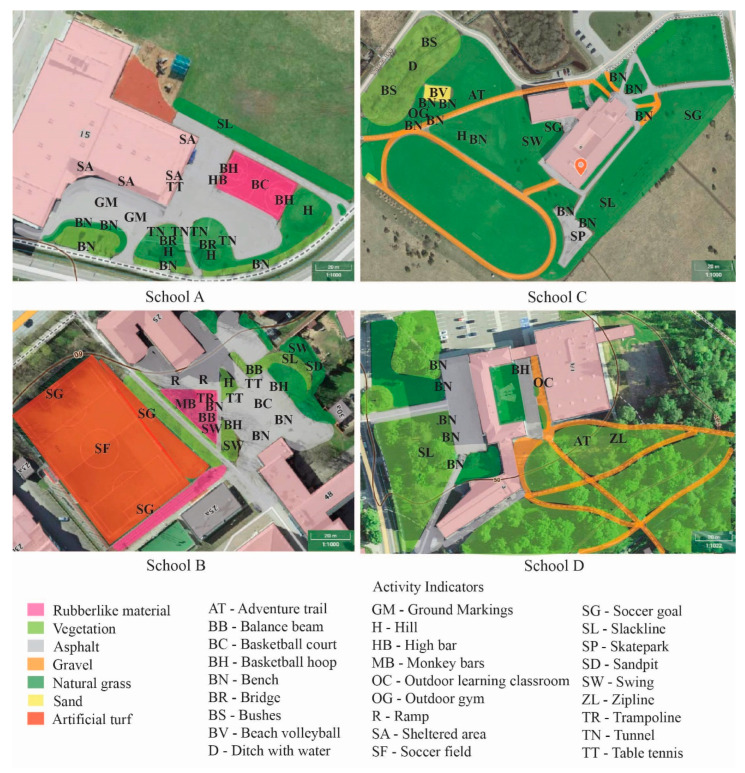
Schoolyards of different schools (descriptions of the schools A, B, C and D are presented in Table 1), showing the characteristics of the design and facilities.

Figure 1 presents the schoolyard mapping cases from each school. School A was located in a city in the southern part of Estonia. The landscape was mainly flat with three artificially made small hills covered with natural grass and some vegetation like trees separating the schoolyard from the road. A large part of the schoolyard was covered with asphalt, which had some markings allowing students to engage in different games and activities. Asphalt also afforded the opportunity for students to ride scooters, bicycles, or skateboards in the schoolyard. In addition, the schoolyard included a basketball court, slackline, a few benches, table tennis, two tunnels going through two of the hills, a bridge connecting two of the hills, and a sheltered area in front of the school. The schoolyard provided opportunities to develop basic motor skills like running, jumping, walking, crawling, balancing, and climbing. Students also had access to different equipment, like balls and rackets, that afforded throwing and catching. Potential hazards of the schoolyard included not having a fence and a road passing right by the schoolyard.

School B was also located in a city in the southern part of Estonia. The landscape was mainly flat with some vegetation like trees and bushes around the sporting areas, natural grass, and artificial surfaces. The multifunctional playground, covered with rubberlike material, included equipment like six swings, monkey bars, four balance beams, and two trampolines in the ground. A large part of the schoolyard was covered with asphalt, including the basketball court and some parking spaces. The asphalt area also included two table tennis tables and two ramps which afforded students to ride their scooters, bicycles, and skateboards. The area covered with natural grass included a slackline, two swings, and a sandpit. School B had an artificial turf soccer field; however, only the older students, not included in this study, used it during recess. Students did not have access to equipment like balls and rackets. Equipment and facilities in the schoolyard afforded running, jumping, balancing, walking, climbing, hanging, throwing, and catching. A potential hazard of the schoolyard was the pedestrian street going through the schoolyard where people also ride their scooters and bicycles.

School C was in a rural area of the northern part of Estonia. The landscape was mainly flat, covered with natural grass with some gravel and asphalt trails in front of and around the school. Behind the school were a hill, bushes, and a ditch with water. This schoolyard also had a beach volleyball court, a low adventure trail, outdoor gym, skatepark with five different size ramps, multiple slacklines, a set of six swings, and multiple soccer goals. Students had access to all kinds of equipment, like different balls, rackets, and even rollerblades. The asphalt road in front of the school afforded students to use rollerblades and ride their scooters, skateboards, and bicycles. The schoolyard had many benches where students can socialise and be together. The track and field stadium was also part of the schoolyard, but it was under construction during the schoolyard mapping process; therefore, students were not allowed to use that area. The schoolyard afforded motor skills like running, walking, jumping, throwing, catching, hanging, climbing, and balancing. A potential hazard of the schoolyard was the road in front of the school which was used by rollerblading students and by the cars of parents bringing their children to school.

School D was in a rural area of the southern part of Estonia. The landscape was mainly flat with a low and long slope with a lot of natural grass and vegetation like trees and bushes. There were gravel trails within the park and some asphalt in front of the school and around the basketball hoop. Asphalt at the front of the school allowed students to ride their bicycles, scooters, and skateboards. An outdoor learning classroom, a low adventure trail with a zipline, and a slackline were also included in the schoolyard. Many benches and tables were located around the school for socialising and outdoor learning. The schoolyard afforded motor skills like running, balancing, walking, climbing, hanging, throwing, and catching. Potential hazards were a parking lot right next to the basketball area and a lake right behind the schoolyard.

### 3.2. Outdoor Recess Observations

In School A, during an outdoor recess for grades four and five, the most used areas of the schoolyard were the basketball court, which had students shooting a basketball for the whole recess, and a slackline area, which also had up to 10 students on or around it for the whole recess (Figure 1, School A–BC, SL). The asphalt area in front of the school was used for riding scooters, running, walking, and playing some racket games (Figure 1, School A–grey area). Two hills, the tunnels, and the bridge were rarely used by students at the school level II throughout recess (Figure 1**,** School A–H, TN, BR). During an outdoor recess for grades one to three, the most popular areas were the hills, tunnels, and the bridge, which had students playing on them throughout the whole recess (Figure 1, School A–H, T, BR). In addition, the slackline had students forming a queue next to it for everybody to be able to use it (Figure 1, School A–SL), and the asphalt area had many students running, walking, and playing games for the whole recess (Figure 1, School A–grey area). Students at school level I also enjoyed natural spaces with a few boys climbing the trees and a big group of students exploring the grass field next to the schoolyard. The less-used area for the students at school level I was the basketball court (Figure 1, School A–BC).

In School B, the most popular area was the multifunctional playground with swings, trampolines, balancing beams, and monkey bars (Figure 1, School B–SW, TR, BB, MB). Trampolines and swings were very popular and were used throughout the whole recess by students from both school levels. Students at school level I used the slackline and played around the natural grass area (Figure 1, School B–SL). An asphalt basketball court was very rarely used, two students rode their bicycles a few times on it and a few boys played some basketball (Figure 1, School B–BC).

In School C, the most used areas were the natural grass area, the asphalt road in front of the school, and the bushes behind the school (Figure 1, School C–BS). In front of the school, many students rode bicycles and were rollerblading (Figure 1, School C–grey area). Students played different ball games and multiple soccer games were happening at the same time (Figure 1, School C–green area). Students playing in the bushes were climbing on the trees, carrying logs, building a shelter, and playing in the water in the ditch (Figure 1, School C–BS, D). The slackline area was also used for the whole recess (Figure 1, School C–SL). Behind the school, students were playing on the hill, using the swings, and playing a soccer game (Figure 1, School C–H, SW, SG). The skatepark, low adventure trail, outdoor gym, and beach volleyball court were used less than other areas in the schoolyard (Figure 1, School C–SP, AT, OG, BV).

In School D, the most used areas were the low adventure trail with a zipline and the asphalt area behind the school (Figure 1, School D–AT, ZL). Many boys rode their scooters and played basketball on the asphalt area (Figure 1, School D–grey area). The low adventure trail was also popular, and four separate groups were formed on the trail. A group of girls kept riding the zipline (Figure 1, School D–AT, ZL). Some students walked through the park to the store. A few boys explored the park (Figure 1, School D–light green area).

### 3.3. Students Preferences for Activities during Outdoor Recess

Three favourite recess activities for boys in both school levels were active games, sports games, and socialising with others (Table 2). Girls enjoy active games, sedentary activities, and socialising with others. When comparing boys and girls, boys enjoy sports games a lot more, whereas girls mentioned engaging in more sedentary activities. Favourite recess activities for school level I were active games, sports games, and sedentary activities. Students at school level II enjoy active games, sports games, and socialising. Students at school level II brought up socialising with others a lot more compared with school level I. A statistically significant difference was found for sports games between genders, and for socialising between school levels.

### 3.4. Physical Activity during Recess

The average amount of time spent in different activity levels of the students participating and not participating in outdoor recess is shown in Table 3. During outdoor recess, students spent 35.5% of the recess time sedentary, compared to 54.7% of the time for the students not participating in outdoor recess. Students participating in outdoor recess engaged in MVPA for 20.4% of recess time, whereas students not participating in outdoor recess spent only 9.5% of the recess time in MVPA.

Girls spent more time being sedentary (40.9% of recess time) in outdoor recess compared to boys (31.2% of recess time). Boys spent more time in MVPA (22.9% of recess time) during outdoor recess compared to girls (17.3% of recess time). Time spent sedentary was significantly lower and time spent in other activity levels in outdoor recess was significantly higher for boys compared with girls (*p* < 0.05; Table 3). Time spent sedentary was significantly higher and time spent in other activity levels was significantly lower when comparing students not participating in outdoor recess with the students who did (*p* < 0.05; Table 3).

The average amount of time spent in different activity levels during recess for school levels I and II are shown in Table 4. Students at school level I spent 35.2% of the outdoor recess in sedentary activity and 17.4% of the time in MVPA, the same indicators for students not participating in outdoor recess were 47.8% and 11.2%, respectively. Students at school level II were sedentary for 35.6% of the outdoor recess and spent 22.2% of the time in MVPA, whereas students who did not participate in outdoor recess spent 58.2% of recess time in sedentary activity and only 8.7% of the time in MVPA. All activity levels except sedentary time were significantly different (*p* < 0.05) for school level II students participating in outdoor recess compared to the students at school level I. All activity levels for school level II students not participating in outdoor recess were significantly different (*p* < 0.05) from students participating in outdoor recess (Table 4).

Comparisons between the average time spent in different activity levels during recess in the four schoolyards are shown in Table 5. Students at School C spent the most time in MVPA (24.4%), whereas time spent in MVPA for other schools ranged from 17–20%. Time spent sedentary was highest in School B (37.5%) and School D (36.8%). The same indicator for the two other schools ranged from 34–35%. School A had the most time spent in light PA (47.8%); the same indicator for all other schools ranged from 41–43%.

## 4. Discussion

The aim of the study was to explore and describe four different schoolyards in Estonia and their affordances for physical activity, and how the students used their schoolyards during outdoor recess. In addition, physical activity levels were assessed among primary school students during recess. The schoolyards varied in size and design. Urban schoolyards had a more constructed environment, whereas rural schoolyards had a more natural environment with a lot of natural grass and trees. All schoolyards included some similarities in facilities like slacklines, ball games, and climbing opportunities. Schoolyard observations showed that students enjoy using natural spaces; however, artificial playgrounds were also regularly used. Students’ questionnaire responses revealed that boys tend to enjoy more sport-related activities, like sports games and movement games, whereas girls engage more in less active activities, like swinging, and indicate the importance of the social aspect of activities. Based on the results of the observation and the accelerometer data, all schoolyards afforded more MVPA during outdoor recess compared to indoor recess, whereas schoolyards with more space per child and a more natural environment generated more MVPA during outdoor recess than smaller artificial schoolyards.

### 4.1. Schoolyard Characteristics and Potential Affordances

The main findings from schoolyard mapping indicate many similarities and differences between the four schoolyards. The landscape of all the schoolyards was characterised by primarily flat topography and surfaces mostly covered by asphalt or natural grass. The total area of the schoolyards ranged from 4581–39,905 m^2^, providing a space of 17–122 m^2^ per student. The urban schools (Schools A and B) had less area per student compared to rural schools (Schools C and D). The open space was typically dominated by asphalt and artificial surfaces in the two urban schools, and by natural grass and vegetation in the two rural schools (see Figure 1 and Table 1). The two urban schoolyards in this study were similar to traditional schoolyards in Estonia, where the focus is on sports fields and artificial surfaces [35]. However, these two schoolyards were fairly new and included other fixed equipment like slacklines, multifunctional playgrounds, and a few hills which also encourage PA among students [28,46,47]. The most versatile schoolyard of the study was that of School C, which provided mostly natural space but also included different play areas, various equipment like rollerblades and a variety of balls, and facilities like swings, slacklines, a low adventure trail, and soccer goals for students to use. The natural environment in School C included bushes and a ditch with water which afforded students to engage in free and fantasy play. Previous studies have emphasised the importance of natural elements for self-initiated PA, creativity, and the social and mental well-being of children [48,49,50].

All four schoolyards afforded a variety of motor skills, inviting mostly locomotor and object control activities. Multifunctional schoolyards are important contributors to children’s motor development, as it challenges different and varied basic movement patterns, stimulates motor competencies, social relationships, and motivation to engage in PA [51,52]. In addition to basic movement skills such as walking, running, and jumping, the asphalt areas in the observed schoolyards facilitated activities with object control skills such as riding scooters and bicycles and rollerblading. Slacklines for balancing and adventure trails or multifunctional playgrounds for climbing, balancing, and hanging were available in all observed schoolyards. Tunnels in School A also afforded crawling and creeping.

### 4.2. Outdoor Recess Observations

The schoolyard is a valuable part of the school for encouraging students’ PA during the school day; therefore, it is important to include areas and facilities that support varied PA and motivate students to participate in PA during outdoor recess. The results showed that different sports fields were less-used areas in the observed schoolyards. If these were used, it was not for over-the-court active play, and students were more likely to shoot a basketball under one hoop or play a modification of a regular soccer game in front of one goal. Therefore, schools with small schoolyards do not have to fit a full-size court into the schoolyard; rather, one multicourt or a few basketball hoops or soccer goals are probably enough [35,53,54]. Therefore, when designing schoolyards, the focus should not be so sport-field-centered.

Natural spaces in observed schoolyards were regularly used. School C had bushes with a ditch next to the school building and students at school level I were actively using it during recess by carrying logs, building a shelter, and playing in the water. Natural spaces stimulate movement literacy like climbing, balancing, coordination, creativity, etc., which are very important competencies for motor development [55]. In urban School A, students at school level I were playing in the large grass field that was right next to the schoolyard during recess. Previous studies have also found that children enjoy natural green spaces more than artificial playgrounds, as they offer different opportunities for multipurpose play and can reduce sedentary behaviour [24,25,56]. Lindemann-Mathies & Köhler [25] found that liking natural grass areas decreased with age, which can account for why students at school level I used and enjoyed playing in natural spaces more during outdoor recess in this study. Pawlowski et al. [57] have found that greenery seemed especially important for girls, as green areas appealed more to social play than competitive sports activities. This is consistent with the observations of this study where a lot of girls seemed to walk around the schoolyard’s greener areas and socialise. Access to green space is also important for the well-being, overall health, and cognitive development of children [19].

School level I students enjoyed using different facilities, with swings, trampolines, monkey bars, adventure trails, and slacklines being the most popular. Powell [21] also found when interviewing 7–10-year-olds in the United Kingdom that the most popular fixed equipment were adventure trails with balance beams and stepping-stones and climbing frames like monkey bars and climbing ropes. Schools that had different equipment, like balls and rackets, available for students to use had more utilization of this equipment. For example, in School C, multiple soccer games occurred at the same time, and in School A and C, some students were shooting a basketball throughout the whole recess, whereas in School B, where equipment was not provided to the students, the basketball court and table tennis were rarely used throughout the recess. Therefore, it can be argued that providing students with various equipment and offering them options for active play could encourage them to be more active throughout recess [58].

### 4.3. Students’ Preferences for Activities during Outdoor Recess

Boys in this study enjoyed more sports games, using natural spaces, and riding scooters/bicycles during outdoor recess, whereas girls preferred socialising with others and activities like sitting, drawing, playing board games, etc. (Table 2). This is in accordance with previous studies that have found that recess time has a highly social function for female students and that they prefer more sedentary activities [20,59,60], whereas male students enjoy more physical activities like basketball, soccer, and other ball games [20,60]. Pawlowski et al. [57] found that for girls it is important to distinguish between facilities for sports and facilities for play. Girls like to be active during recess; however, they were more likely to mention activities which were not sport related, like challenging themselves, climbing, dancing, and engaging in active roleplay. In this study, girls also mentioned enjoying more active games, like tag, hide and seek, jump rope, etc., instead of traditional sport-related ball games. Providing schoolyards with gender-specific equipment and activities during recess could be a solution for encouraging girls to be more physically active during recess [61] and might more effectively provide them with opportunities to socialise during this time [59]. Comparing the responses of school levels I and II, the two most-mentioned activities for both school levels were active games and sports games. The following category for school level I was sedentary activities, whereas school level II mentioned socialising with others. These findings are consistent with Holmes [20] who observed that all students from kindergarten to grade eight enjoyed playing active and sports games, whereas socialising with peers was less common among younger students and was a preferred activity of children in grades three to eight. Therefore, boys and girls seem to have different needs, and gender differences seem to increase with age, and younger children prefer more playful and varied activities, whereas sports and movement games become more popular with age [62].

### 4.4. Physical Activity during Recess

Consistent evidence suggests that the schoolyard is an important source for accumulating daily MVPA in children and youth [47,63,64]. Students in this study who attended outdoor recess had more time spent in MVPA during recess and significantly less sedentary time when compared to the students who did not attend outdoor recess. The difference was particularly noticeable at school level II, where students not attending outdoor recess had more than 50% less time spent in MVPA than students who participated in outdoor recess. Similar to the results of this study, Tran et al. [17] found that more MVPA was achieved during outdoor recess compared to indoor recess. Students in this study spent about 21% of the outdoor recess time in MVPA and boys were more active than girls (Table 3). This is consistent with previous studies which have also found boys to be more active during outdoor recess [17,46,59,63,65]. A possible explanation for this might be that boys also engage more in competitive sports, whereas girls use areas that promote social interaction [47,53,59]. Time spent in MVPA during outdoor recess in observed schools is similar or a bit lower compared to the results of other studies carried out in the USA [28,66], the Netherlands [63], and Germany [67], which recorded time spent in MVPA during recess between 25.8–48.8%. A study carried out in Sweden by Pagels et al. [65] found that large play fields and woodland areas facilitated significantly more time spent in MVPA during outdoor time compared to schoolyards with smaller areas. These results are consistent with the findings of this study, as the two schools (School C and D) with the largest and most natural schoolyards had the most time spent in MVPA during outdoor recess, whereas School B, an urban school with a smaller schoolyard, had the most sedentary time.

The sedentary time of school level I and school level II during outdoor recess did not show a significant difference; however, among students not attending outdoor recess, a significant difference was found for sedentary time when comparing school level I and school level II (Table 3). Students in school level II not attending outdoor recess had significantly more sedentary time compared with students in school level I. This could imply that younger students also find active play opportunities during indoor recess [68]. Students at school level I participating in outdoor recess spent significantly more time in light PA, whereas those at school level II spent significantly more time in MVPA. This finding is contrary to that of Pagels et al. [65], who found that in four Swedish schools, 5th and 8th graders were less physically active than 2nd graders during outdoor play. This result may be explained by the fact that younger students in this study reported enjoying active activities like climbing and swinging, whereas older students like to play sports and active games that require higher-intensity PA (Table 2).

Outdoor play is a great source for increasing children’s everyday MVPA. However, in the current study, the level of MVPA throughout the school day was still quite low compared to the WHO’s recommendation [1]. According to the accelerometer data of this study on outdoor recess, it seems that children in Estonia need to acquire most of their MVPA after school hours, although they spend the majority of their waking hours at school. Regardless of gender or school level, students in this study obtained about 12% of their daily MVPA during outdoor recess. Other countries, like Norway, have shown that children can obtain close to the recommended 60 min of MVPA during school hours if the schoolyard is varied and multifunctional [29]. Therefore, Estonia is taking steps in the right direction to provide children with opportunities to meet the 60 min MVPA recommendation during the school day.

### 4.5. Strengths and Limitations

This study has strengths and limitations that should be discussed. The strength of this study is that it was the first study that described and observed different schoolyards and their affordances, while at the same time measuring the PA during outdoor and indoor recess, in Estonia. The results of the study can be shared with stakeholders to suggest what type of schoolyards and daily schedules encourage varied PA among all students. One of the limitations of this study was that it was not possible to observe only the students wearing the accelerometers during outdoor recess, as all students had the opportunity to go outside during the same recess. In addition, students wore the accelerometers for seven days, but the observations took place on the first two days and not throughout the whole study period. Another limitation of the study is that the observations took place in autumn in two urban schools, and in spring in two rural schools. Pagels et al. [65] found that the outdoor temperature significantly impacted MVPA during outdoor play, therefore, future research should observe the outdoor recesses in the same school in different seasons to obtain more objective results of PA levels.

### 4.6. Practical Implications

The results of this study provide insight into what types of schoolyards invite students to engage in more MVPA and how outdoor recess should be implemented to increase MVPA engaged in during the school day. School leaders, together with local and national municipalities, can use this insight when designing new or reconstructing old schoolyards. The focus should be on providing various equipment, facilities, and activities for all students based on their gender, age, and skills. In addition, stakeholders can use the PA data from this study in order to implement outdoor recess in the school curriculum. The PA results of this study showed that students participating in outdoor recess engaged in more MVPA than students who did not; therefore, providing outdoor recess is especially important for students who are less active after school. Generalised information provided in this article can be used in a wider, not just in an Estonian, context as outdoor recess and schoolyard characteristics are universal factors in students’ PA.

## 5. Conclusions

The results showed that implementing outdoor recess in schools is important for students’ PA. All observed schoolyards varied in space, landscape, and vegetation, encouraging MVPA during outdoor recess. However, schoolyards that had more natural space, various equipment for students to use, and some fixed equipment facilitated even more MVPA. Observations and questionnaire responses indicated that boys enjoy more sport-related activities, whereas girls like more sedentary activities and emphasise the social factor of activities. The accelerometer results revealed that students had significantly higher MVPA during outdoor recess compared to indoor recess at both school levels, while the amount of sedentary time was significantly higher in indoor recess. Schoolyards and outdoor recess give an opportunity for students to be more active, therefore contributing towards their daily PA. Furthermore, outdoor recess for some students might be the only opportunity to be active during the day. Full-size sports fields do not have to be part of the schoolyard to invite students to move; just a soccer goal or a basketball hoop can be enough to provide students with opportunities to be active.

## Figures and Tables

**Table 1:** Schoolyard characteristics in four schools in Estonia.

School	Schoolyard Area, Pupils (*n*), and Area/Child	Landscape: Topography and Vegetation	Surface	Dominating Main Characteristics
School A	4581 m^2^ *n* = 216 21 m^2^	Mostly flat, two artificial hills, open. Vegetation: natural grass, trees.	Asphalt, natural grass, rubberlike material	Open asphalt area, opportunity to bike. Basketball court, two hills connected with a bridge.
School B	15,406 m^2^ *n* = 909 17 m^2^	Flat, open. Vegetation: some natural grass and big trees.	Asphalt, natural grass, rubberlike material	Basketball court, multifunctional playground.
School C	39,905 m^2^ *n* = 327 122 m^2^	Flat, open. Vegetation: some trees and bushes, and a ditch with water.	Natural grass, gravel, asphalt, sand	Open grass area, slacklines, skatepark, opportunity to bike & rollerblade.
School D	34,706 *n* = 764 45 m^2^	Mostly flat, low, and long slope. Vegetation: a lot of big trees and bushes.	Natural grass, gravel, asphalt	Park, adventure trail with zipline, opportunity to bike.

**Table 2:** Outdoor recess activity preference shown in percentage (%), comparison between genders and school levels.

Recess Activities	Boys (%)	Girls (%)	School Level I (%)	School Level II (%)
Active games (i.e., tag)	62.9	64.7	73.1	58.9
Sports games (i.e., basketball, soccer, etc.)	72.2 *	28.3	46.3	51.9
Walking	21.7	30.3	17.9	30.2
Socialising	22.7	33.3	10.5 ^#^	37.2
Sedentary activities	12.4	35.4	25.4	23.3
Running	9.3	11.1	10.5	10.1
Climbing	9.3	15.2	16.4	10.1
Facilities (i.e., swing, trampoline, etc.)	16.5	13.1	20.9	11.6
Slackline	3.1	16.2	11.9	8.5
Using a natural space	13.4	2.0	17.9	37.2
Riding a bicycle, scooter/rollerblading	15.5	4.0	11.9	8.5
Singing/dancing	1.0	4.0	1.5	3.1
Studying	5.2	8.1	3.0	8.5
Imaginary games	1.0	3.0	6.0	0.0
Standing	2.1	0.0	1.5	0.8
Eating	0.0	4.0	0.0	3.1
Working out	0.0	1.0	0.0	0.8

* Significantly different from girls. ^#^ Significantly different from school level II.

**Table 3:** Differences in time spent in different activity levels (mean ± SE) during outdoor/indoor recess.

Variable	Participated in Outdoor Recess			Participated in Indoor Recess		
	Boys	Girls	Total	Boys	Girls	Total
Participation cases ^&^	318	260	578	164	206	370
Sedentary PA (min/day)	11.5 ± 0.4	14.9 ± 0.5 ^#^	13.0 ± 0.3	19.2 ± 0.6 *	20.8 ± 0.6 *	20.1 ± 0.4 *
Light PA (min/day)	16.9 ± 0.3	15.5 ± 0.4 ^#^	16.3 ± 0.2	13.8 ± 0.5 *	12.8 ± 0.4 *	13.2 ± 0.3 *
Moderate PA (min/day)	5.4 ± 0.2	4.0 ± 0.2 ^#^	4.7 ± 0.1	2.8 ± 0.3 *	2.3 ± 0.2 *	2.5 ± 0.2 *
Vigorous PA (min/day)	3.2 ± 0.2	2.6 ± 0.2 ^#^	2.9 ± 0.1	1.1 ± 0.2 *	1.1 ± 0.2 *	1.1 ± 0.2 *
MVPA (min/day)	8.6 ± 0.3	6.6 ± 0.3 ^#^	7.7 ± 0.2	3.9 ± 0.4 *	3.4 ± 0.4 *	3.6 ± 0.3 *

Adjusted for the length of outdoor recess. ^&^ The number of children indicating participation in outdoor or indoor recess was counted for each day and was summed as total participation in outdoor or indoor recess. ^#^ Significantly different from males. * Significantly different from participation in outdoor recess.

**Table 4:** Differences in time spent in different activity levels between school levels I and II (mean ± SE) during outdoor/indoor recess.

Variable	Participated in Outdoor Recess		Participated in Indoor Recess	
	School Level I	School Level II	School Level I	School Level II
Participation cases ^&^	220	358	150	220
Sedentary PA (min/day)	12.9 ± 0.6	13.1 ± 0.4	17.4 ± 0.7 *	21.5 ± 0.5 *^#^
Light PA (min/day)	18.3 ± 0.4	15.2 ± 0.3 ^#^	15.0 ± 0.5 *	12.2 ± 0.4 *^#^
Moderate PA (min/day)	3.8 ± 0.2	5.2 ± 0.2 ^#^	3.1 ± 0.3	2.3 ± 0.2 *^#^
Vigorous PA (min/day)	1.9 ± 0.2	3.5 ± 0.2 ^#^	1.4 ± 0.3	1.1 ± 0.2 *
MVPA (min/day)	5.7 ± 0.4	8.7 ± 0.3 ^#^	4.5 ± 0.5	3.3 ± 0.3 *

Adjusted for the length of outdoor recess. ^&^ The number of children indicating participation in outdoor or indoor recess was counted for each day and was summed as total participation in outdoor or indoor recess. ^#^ Significantly different from school level I. * Significantly different from participation in outdoor recess.

**Table 5:** Differences in time spent in different activity levels between the four schools (mean ± SE) during outdoor/indoor recess.

Variable	School A		School B		School C		School D	
	Participated in Outdoor Recess	Participated in Indoor Recess	Participated in Outdoor Recess	Participated in Indoor Recess	Participated in Outdoor Recess	Participated in Indoor Recess	Participated in Outdoor Recess	Participated in Indoor Recess
Participation cases ^&^	205	27	81	138	204	55	88	150
Sedentary PA (min/day)	12.8 ± 0.5	19.8 ± 1.5 *	14.2 ± 0.9	20.3 ± 0.7 *	13.0 ± 0.6	25.3 ± 1.1 *^,1,2^	12.9 ± 0.9	17.1 ± 0.8 *^,2,3^
Light PA (min/day)	17.8 ± 0.4	14.4 ± 1.1 *	15.5 ± 0.6 ^1^	13.6 ± 0.5 *	14.9 ± 0.4 ^1^	8.6 ± 0.7 *^,1,2^	16.5 ± 0.7 ^3^	15.1 ± 0.6 ^3^
Moderate PA (min/day)	4.1 ± 0.2	2.0 ± 0.6 *	4.6 ± 0.3	2.2 ± 0.3 *	5.4 ± 0.2^12^	1.9 ± 0.4 *	4.8 ± 0.4	3.3 ± 0.3 *^,1,2,3^
Vigorous PA (min/day)	2.3 ± 0.2	0.9 ± 0.5 *	2.4 ± 0.3	0.9 ± 0.2 *	3.7 ± 0.2 ^1,2^	1.1 ± 0.4 *	2.7 ± 0.3 ^3^	1.5 ± 0.3 *
MVPA (min/day)	6.4 ± 0.4	2.9 ± 0.9 *	7.0 ± 0.6	3.1 ± 0.4 *	9.1 ± 0.4 ^1,2^	3.0 ± 0.7 *	7.6 ± 0.6 ^3^	4.8 ± 0.5 *^,2,3^

Adjusted for length of outdoor recess. ^&^ The number of children indicating participation in outdoor or indoor recess was counted for each day and was summed as total participation in outdoor or indoor recess. * Significantly different from participation in outdoor recess. ^1^ Significantly different from School A. ^2^ Significantly different from School B. ^3^ Significantly different from School C.

## Data Availability

The data presented in this study are available on request from E.M.

## References

[1] World Health Organization (2020). WHO Guidelines on Physical Activity and Sedentary Behaviour: Web Annex: Evidence Profiles.

[2] Zeng N., Ayyub M., Sun H., Wen X., Xiang P., Gao Z. (2017). Effects of physical activity on motor skills and cognitive development in early childhood: A systematic review. BioMed Res. Int..

[3] Poitras J.V., Gray C.E., Borghese M.M., Carson V., Chaput J.P., Janssen I., Katzmarzyk P.T., Pate R.R., Gorber S.C., Kho M.E. (2016). Systematic review of the relationship between objectively measured physical activity and health indicators in school-aged children and youth. Appl. Physiol. Nutr. Metab..

[4] De Greeff J.W., Bosker R.J., Oosterlaan J., Visscher C., Hartman E. (2018). Effects of physical activity on executive functions, attention and academic performance in preadolescent children: A meta-analysis. J. Sci. Med. Sport.

[5] Muntaner-Mas M., Martinez-Gomez D., Castro-Piñero J., Fernandez-Santos J.R., Salmon J., Veiga O.L., Esteban-Cornejo I. (2021). Objectively measured physical activity and academic performance in school-aged youth: The UP&DOWN longitudinal study. Scand. J. Med. Sci. Sports.

[6] Rodriguez-Ayllon M., Cadenas-Sánchez C., Estévez-López F., Muñoz N.E., Mora-Gonzalez J., Migueles J.H., Molina-García P., Henriksson H., Mena-Molina A., Martínez-Vizcaíno V. (2019). Role of physical activity and sedentary behavior in the mental health of preschoolers, children and adolescents: A systematic review and meta-analysis. Sports Med..

[7] Mäestu E., Jürimäe J., Kull M., Koka A., Pihu M., Riso E.M., Kais K., Tilga H., Mäestu J. (2022). Estonian’s 2021 Report Card on Physical Activity for Children and Youth.

[8] Oja L., Pikksööt J., Aasvee K., Haav A., Kasvandik L., Kukk M., Kukke K., Rahno J., Saapar M., Vorobjov S. (2019). Health Behaviour in School-Aged Children (HBSC). Estonian Survey Report.

[9] Sanchez S.P., Gallego D.I. (2021). Evidence-based overview of accelerometer-measured physical activity during school recess: An updated systematic review. Int. J. Environ. Res. Public Health.

[10] Ekelund U., Luan J., Sherar L.B., Esliger D.W., Griew P., Cooper A., International Children’s Accelerometry Database (ICAD) Collaborators (2012). Moderate to vigorous physical activity and sedentary time and cardiometabolic risk factors in children and adolescents. JAMA.

[11] Dobbins M., Husson H., DeCorby K., LaRocca R.L. (2013). School-based physical activity programs for promoting physical activity and fitness in children and adolescents aged 6 to 18. Cochrane Database Syst. Rev..

[12] Naylor P.J., McKay H.A. (2009). Prevention in the first place: Schools a setting for action on physical inactivity. Br. J. Sports Med..

[13] Bell A., Dyment J. (2006). Grounds for Action: Promoting Physical Activity Through School Ground Greening in Canada.

[14] Blaes A., Ridgers N.D., Aucouturier J., Van Praagh E., Berthoin S., Baquet G. (2013). Effects of a playground marking intervention on school recess physical activity in French children. Prev. Med..

[15] Ishii K., Shibata A., Sato M., Oka K. (2014). Recess physical activity and perceived school environment among elementary school children. Int. J. Environ. Res. Public Health.

[16] Andersen H., Klinker C.D., Toftager M., Pawlowski C., Schipperijn J. (2015). Objectively measured differences in physical activity in five types of schoolyard area. Landsc. Urban Plan..

[17] Tran I., Clark B.R., Racette S.B. (2013). Physical activity during recess outdoors and indoors among urban public school students, St. Louis, Missouri, 2010–2011. Prev. Chronic Dis..

[18] Hyndman B., Benson A., Ullah S., Telford A. (2014). Evaluating the effects of the Lunchtime Enjoyment Activity and Play (LEAP) school playground intervention on children’s quality of life, enjoyment and participation in physical activity. BMC Public Health.

[19] McCormick R. (2017). Does access to green space impact the mental well-being of children: A systematic review. J. Pediatr. Nurs..

[20] Holmes R. (2012). The outdoor recess activities of children at an urban school: Longitudinal and intraperiod patterns. Am. J. Play.

[21] Powell E., Woodfield L.A., Nevill A.A. (2016). Children’s physical activity levels during primary school break times: A quantitative and qualitative research design. Eur. Phys. Educ. Rev..

[22] Armstrong G., Maitland C., Lester L., Trost S., Trapp G., Boruff B., Al Marzooqi M., Christian H. (2019). Associations between the home yard and preschoolers’ outdoor play and physical activity. Public Health Res. Pract..

[23] Chawla L., Keena K., Pevec I., Stanley E. (2014). Green schoolyards as havens from stress and resources for resilience in childhood and adolescence. Health Place.

[24] Raney M.A., Hendry C.F., Yee S.A. (2019). Physical activity and social behaviors of urban children in green playgrounds. Am. J. Prev. Med..

[25] Lindemann-Matthies P., Köhler K. (2019). Naturalized versus traditional school grounds: Which elements do students prefer and why?. Urban For. Urban Green..

[26] Baquet G., Aucouturier J., Gamelin F.X., Berthoin S. (2018). Longitudinal follow-up of physical activity during school recess: Impact of playground markings. Front. Public Health.

[27] Parrish A.M., Okely A.D., Stanley R.M., Ridgers N.D. (2013). The effect of school recess interventions on physical activity: A systematic review. Sports Med..

[28] Anthamatten P., Brink L., Kingston B., Kutchman E., Lampe S., Nigg C. (2014). An assessment of schoolyard features and behaviour patterns in children’s utilization and physical activity. J. Phys. Act. Health.

[29] Kjønniksen L., Wiium N., Fjørtoft I. (2022). Affordances of school ground environments for physical activity: A case study on 10- and 12-year-old children in a Norwegian primary school. Front. Public Health.

[30] Brustio P.R., Moisè P., Marasso D., Miglio F., Rainoldi A., Boccia G. (2018). Feasibility of implementing an outdoor walking break in Italian middle schools. PLoS ONE.

[31] Van Dijk-Wesselius J.E., Maas J., Hovinga D., van Vugt M., van den Berg A.E. (2018). The impact of greening schoolyards on the appreciation, and physical, cognitive and social-emotional well-being of schoolchildren: A prospective intervention study. Landsc. Urban Plan..

[32] Kuo M., Browning M.H.E.M., Sachdeva S., Lee K., Westphal L. (2018). Might school performance grow on trees? Examining the link between “greenness” and academic achievement in urban, high-poverty schools. Front. Psychol..

[33] Browning M.H.E.M., Rigolon A. (2019). School green space and its impact on academic performance: A systematic literature review. Int. J. Environ. Res. Public Health.

[34] Bikomeye J.C., Balza J., Beyer K.M. (2021). The impact of schoolyard greening on children’s physical activity and socioemotional health: A systematic review of experimental studies. Int. J. Environ. Res Public Health.

[35] Rutkauskaite R., Gisladottir T., Pihu M., Kjønniksen L., Lounassalo I., Huovinen T., Gruodyte-Raciene R., Visagurskiene K., Olafson O., Kull M. (2021). Schoolyard affordances for physical activity: A pilot study in 6 Nordic–Baltic countries. Sustainability.

[36] Mooses K., Vihalemm T., Uibu M., Mägi K., Korp L., Kalma M., Mäestu E., Kull M. (2021). Developing a comprehensive school based physical activity program with flexible design—From pilot to national program. BMC Public Health.

[37] Klementi K., Koov K., Org T. (2019). Muutuv Kooliruum. Eesti Arhitektide Liidu Juhend Tänapäevast Õpikäsitust Toetava Koolikeskkonna Kavandamiseks. Estonian Association of Architects. https://www.digar.ee/arhiiv/nlib-digar:419647.

[38] Gibson J.J. (1979). The theory of affordances. The Ecological Approach to Visual Perception.

[39] Heft H. (1988). Affordances of children’s environments: A functional approach to environmental design. Child Environ. Q..

[40] World Medical Association (2013). World Medical Association Declaration of Helsinki: Ethical principles for medical research involving human subjects. JAMA.

[41] Kristiansand Municipality and University of Agder (2006). Aktiv Ute. Kartlegging av Skolegårder. (“Active Outdoors”. Mapping Schoolyards, Design and Instructions).

[42] Estonian Land Board. X-GIS(2). https://xgis.maaamet.ee/maps/XGis?app_id=UU82A&user_id=at&LANG=1&WIDTH=1140&HEIGHT=772&zlevel=0,552500,6505000.

[43] Adobe Inc (2019). Adobe Illustrator. https://adobe.com/products/illustrator.

[44] McKenzie T.L. (2012). System for Observing Play and Leisure Activity in Youth (SOPLAY). Meas. Instrum. Database Soc. J. Sport. Sci..

[45] Evenson K.R., Catellier D.J., Gill K., Ondrak K.S., McMurray R.G. (2008). Calibration of two objective measures of physical activity for children. J. Sports Sci..

[46] Van Kann D.H., de Vries S.I., Schipperijn J., de Vries N.K., Jansen M.W., Kremers S.P. (2016). Schoolyard characteristics, physical activity, and sedentary behavior: Combining GPS and accelerometry. J. Sch. Health.

[47] Graham M., Wright M., Azevedo L.B., Macpherson T., Jones D., Innerd A. (2021). The school playground environment as a driver of primary school children’s physical activity behaviour: A direct observation case study. J. Sports Sci..

[48] Dowdell K., Gray T., Malone K. (2011). Nature and its influence on children’s outdoor play. J. Outdoor Environ. Educ..

[49] Kiewra C., Veselack E.M. (2016). Playing with Nature: Supporting Preschoolers’ Creativity in Natural Outdoor Classrooms. Int. J. Early Child. Environ. Educ..

[50] Engemann K., Bøcker C., Pedersen L.A., Tsirogiannis C., Bo Mortensen P., Svenning J.C. (2019). Residential green space in childhood is associated with lower risk of psychiatric disorders from adolescence into adulthood. Proc. Natl. Acad. Sci. USA.

[51] Tremblay M.S., Gray C., Babcock S., Barnes J., Bradstreet C.C., Carr D., Chabot G., Choquette L., Chorney D., Collyer C. (2015). Position statement on active outdoor play. Int. J. Environ. Res. Public Health.

[52] Tortella P., Fumagalli G. (2021). Urban outdoor movement education: A playground to promote physical activity. The case of the “Primo Sport 0246” playground. J. Phys. Educ. Sport.

[53] Andersen H., Christiansen L., Pawlowski C., Schipperijn J. (2019). What we build makes a difference—Mapping activating schoolyard features after renewal using GIS, GPS and accelerometers. Landsc. Urban Plan..

[54] Fjørtoft I., Löfman O., Halvorsen Thorén K. (2010). Schoolyard physical activity in 14-year-old adolescents assessed by mobile GPS and heart rate monitoring analysed by GIS. Scand. J. Public Health.

[55] Fjørtoft I. (2004). Landscape as Playscape. The effect of natural environments on children’s play and motor development. Child. Youth Environ..

[56] Dankiw K.A., Tsiros M.D., Baldock K.L., Kumar S. (2020). The impacts of unstructured nature play on health in early childhood development: A systematic review. PLoS ONE.

[57] Pawlowski C.S., Veitch J., Andersen H.B., Ridgers N.D. (2019). Designing activating schoolyards: Seen from the girls’ viewpoint. Int. J. Environ. Res. Public Health.

[58] Verstraete S.J., Cardon G.M., De Clercq D.L., De Bourdeaudhuij I.M. (2006). Increasing children’s physical activity levels during recess periods in elementary schools: The effects of providing game equipment. Eur. J. Public Health.

[59] Pawlowski C.S., Andersen H.B., Troelsen J., Schipperijn J. (2016). Children’s physical activity behavior during school recess: A pilot study using GPS, accelerometer, participant observation, and go-along interview. PLoS ONE.

[60] London R.A. (2022). It is not called recess anymore: Breaktime in middle school. J. Sch. Health.

[61] Haapala H.L., Hirvensalo M.H., Laine K., Laakso L., Hakonen H., Lintunen T., Tammelin T.H. (2017). Differences in physical activity at recess and school-related social factors in four Finnish lower secondary schools. Health Educ. Res..

[62] Kjønniksen L., Torsheim T., Wold B. (2008). Tracking of leisure-time physical activity during adolescence and young adulthood: A 10 year follow-up study. Int. J. Behav. Nutr. Phys. Act..

[63] Dessing D., Pierik F.H., Sterkenburg R.P., van Dommelen P., Maas J., de Vries S.I. (2013). Schoolyard physical activity of 6-11 year old children assessed by GPS and accelerometry. Int. J. Behav. Nutr. Phys. Act.

[64] Klinker C.D., Schipperijn J., Christian H., Kerr J., Ersbøll A.K., Troelsen J. (2014). Using accelerometers and global positioning system devices to assess gender and age differences in children’s school, transport, leisure and home based physical activity. Int. J. Behav. Nutr. Phys. Act..

[65] Pagels P., Raustorp A., De Leon A.P., Mårtensson F., Kylin M., Boldemann C. (2014). A repeated measurement study investigating the impact of school outdoor environment upon physical activity across ages and seasons in Swedish second, fifth and eighth graders. BMC Public Health.

[66] Ridgers N., Saint-Maurice P.F., Welk G.J., Siahpush M., Huberty J. (2011). Differences in physical activity during school recess. J. Sch. Health.

[67] Kobel S., Kettner S., Erkelenz N., Kesztyüs D., Steinacker J.M. (2015). Does a higher incidence of break times in primary schools result in children being more physically active?. J. Sch. Health.

[68] Wollersheim Shervey S., DiPerna J. (2017). Engagement in physical activity during recess: Gender and grade level differences in the elementary grades. J. Phys. Act. Health.

